# Supplementary material for: Detailed molecular and epigenetic characterization of the pig IPEC-J2 and chicken SL-29 cell lines
Source: iScience. 2023 Feb 20;26(3):106252. doi: 10.1016/j.isci.2023.106252 (PMC10018572; doi:10.1016/j.isci.2023.106252)
Supplement: Data S2. Complete homer output for identified motifs in Chicken SL-29, related to Tables 5 and 6 — Homer motif analysis results for histone modifications H3K4me1, H3K4me3, H3K27ac, enhancers, and ATAC-seq of chicken SL-29 cell line. Parameters for possible false positives is as mentioned earlier for S5. [file mmc3.zip › Data_S2/S6/Chicken_SL_29/peak_files_CTCF/homerResults/motif1.similar.html]

motif1

## Information for motif1

T
C
A
G
G
A
C
T
C
A
T
G
G
C
T
A
A
T
C
G
G
C
A
T
G
T
A
C
G
T
C
A
A
G
C
T
G
A
T
C
  
Reverse Opposite:  

C
T
A
G
T
C
G
A
C
A
G
T
A
C
T
G
C
G
T
A
T
A
G
C
C
G
A
T
G
T
A
C
C
T
G
A
A
G
T
C
  

|  |  |
| --- | --- |
| p-value: | 1e-1238 |
| log p-value: | -2.852e+03 |
| Information Content per bp: | 1.668 |
| Number of Target Sequences with motif | 5590.0 |
| Percentage of Target Sequences with motif | 18.96% |
| Number of Background Sequences with motif | 1763.8 |
| Percentage of Background Sequences with motif | 6.06% |
| Average Position of motif in Targets | 150.8 +/- 80.9bp |
| Average Position of motif in Background | 148.9 +/- 87.8bp |
| Strand Bias (log2 ratio + to - strand density) | -0.0 |
| Multiplicity (# of sites on avg that occur together) | 1.18 |
| Motif File: | file (matrix) reverse opposite |

### Similar de novo motifs found

|  |  |  |  |  |  |  |  |
| --- | --- | --- | --- | --- | --- | --- | --- |
| Rank | Match Score | Redundant Motif | P-value | log P-value | % of Targets | % of Background | Motif file |
| 1 | 0.955 | A C G T A C T G G C T A A T C G A C G T G T A C C G T A A G C T | 1e-1139 | -2623.551079 | 17.31% | 5.47% | motif file (matrix) |
| 2 | 0.969 | G C T A C T A G A C T G G T C A C G T A T G C A A C G T A C T G C G T A T A C G A G C T T G A C C G T A | 1e-984 | -2266.210064 | 17.17% | 5.95% | motif file (matrix) |
| 3 | 0.926 | T G C A A C G T A C T G C G T A G A T C C G A T A G T C C T G A | 1e-879 | -2025.662382 | 17.98% | 6.89% | motif file (matrix) |
| 4 | 0.943 | C G A T A C T G C G T A T A C G C G A T G T A C C G T A C A G T T A C G T G A C | 1e-754 | -1737.437962 | 25.84% | 13.02% | motif file (matrix) |
| 5 | 0.855 | A C G T A C G T C G T A A T G C A C G T G T A C C G T A A T C G | 1e-445 | -1025.855221 | 12.71% | 5.69% | motif file (matrix) |
| 6 | 0.730 | T C G A C A G T C A T G C G T A A T G C G C A T T G C A C G T A G A C T T C G A A G T C G C A T A C G T | 1e-83 | -192.392520 | 1.55% | 0.54% | motif file (matrix) |
| 7 | 0.697 | A C G T A C T G A C G T A G C T A C T G C G T A A C G T A C G T A G T C C G T A | 1e-59 | -137.036226 | 0.58% | 0.12% | motif file (matrix) |
